# Adaptive Broadcasting Mechanism for Bandwidth Allocation in Mobile Services

**DOI:** 10.1155/2014/735457

**Published:** 2014-06-26

**Authors:** Gwo-Jiun Horng, Chi-Hsuan Wang, Chih-Lun Chou

**Affiliations:** ^1^Department of Computer Science and Information Engineering, Southern Taiwan University of Science and Technology, Tainan 710, Taiwan; ^2^Department of Electrical Engineering, National Cheng Kung University, Taiwan; ^3^Department of Information and Telecommunications Engineering, Ming Chuan University, Taiwan

## Abstract

This paper proposes a tree-based adaptive broadcasting (TAB) algorithm for data dissemination to improve data access efficiency. The proposed TAB algorithm first constructs a broadcast tree to determine the broadcast frequency of each data and splits the broadcast tree into some broadcast wood to generate the broadcast program. In addition, this paper develops an analytical model to derive the mean access latency of the generated broadcast program. In light of the derived results, both the index channel's bandwidth and the data channel's bandwidth can be optimally allocated to maximize bandwidth utilization. This paper presents experiments to help evaluate the effectiveness of the proposed strategy. From the experimental results, it can be seen that the proposed mechanism is feasible in practice.

## 1. Introduction

Mobile web services are a new generation of web services accessible to mobile clients through the air in support of anytime-and-anywhere access to services [1, 2]. Furthermore, owing to the characteristics of wireless environments including device mobility, scarce bandwidth, and limited battery power, accessing services in wireless-oriented service environments has become an emerging challenge to the data-management and telecommunication communities [3].

In essence, there are two fundamental modes for data-service dissemination in a wireless region: the broadcasting mode and the on-demand mode [4]. In a broadcasting mode, data is broadcast periodically to mobile devices according to a broadcast program in the region [5–14]. To fetch a data record, mobile clients have to wait until the target data appears on the broadcast channel. In this way, a broadcast-based system can serve thousands of mobile users simultaneously, since the broadcast cost is identical regardless of the number of users. The other data dissemination mode is the on-demand mode. This mode is similar to the traditional client-server approach. In the on-demand mode, a mobile node first sends its query on an uplink channel and the server sends the requested data to the client through the downlink channel. In this paper, we consider data disseminated in a broadcast-based wireless environment.

In the literature, access efficiency and energy consumption are two issues of concern in assessing the performance of wireless communication systems [4, 15]. Access efficiency can be evaluated by access time, which means the time that has elapsed from the moment a client requests data to the moment the client retrieves the target item. Energy consumption concerns the battery power consumed by the client to retrieve the requested data, and it can be quantified according to tune-in time [15], in other words, according to the amount of time the mobile device stays active “listening” to the broadcast channel.

The plain-broadcast scheme is the simplest approach to generating data-broadcast programs and has been adopted in earlier research [3, 16]. Using this approach, the server broadcasts all data records in a round robin manner. Therefore, this method is easily implemented. Furthermore, since the plain-broadcast scheme treats all data items equally, the average waiting time for each packet of data equals half of the overall broadcast period. As a result, it is clear that this scheme is not feasible for cases in which data-access frequencies are not uniform.

An alternative data dissemination mechanism is the broadcast disks scheme, which permits data items to be broadcast with different frequencies [5]. This algorithm first divides data items into a few groups (i.e., disks) such that data items with similar popularity are assigned to the same disks. Afterwards, it determines the rotation speed of each disk according to the popularity of data items. In this way, one can construct a broadcast program that adjusts the trade-off between the access time of hot data and that of cold data.

In addition to access efficiency, power conservation is critical for mobile nodes owing to limited battery capacities [17–19]. To facilitate power saving, it is necessary for mobile devices to support two operation modes: the active mode and the doze mode [20]. Mobile clients normally operate in the active mode, and they can switch to the energy-saving doze mode when mobile devices become idle. Thus, keeping mobile devices in the doze mode for as long as possible could be achieved through the application of an air index technique.

By broadcasting the arrival time of data items to clients, mobile devices can stay in the doze mode until the requested data arrives. In this way, the tune-in time can be reduced to the initial index probe time plus the data-retrieval time. At present, several research efforts have addressed reducing the initial probe time [15, 21–26]. These studies complement our work in different aspects.

In this paper, we investigate the effects of data-access frequency and data size on access efficiency. And we propose the tree-based adaptive broadcasting (TAB) algorithm for skewed-data-access to generate an efficient broadcast cycle. The TAB algorithm first generates a broadcast tree to determine the broadcast frequency of each data record. After that, the broadcast tree is split into broadcast wood to balance the interbroadcast time of successive copies of data. In order to reduce the tune-in time, we further separate one individual channel from the broadcast channel to broadcast index packets.

The rest of this paper is organized as follows.  Section 2 introduces related data-broadcast research. The system architecture used throughout this paper is presented as well.  Section 3 discusses the proposed TAB algorithm and its role in improving data-access latency.  Section 4 establishes an analytical model for optimizing index-channel and data-channel bandwidth allocation.  Section 5 discusses the proposed dynamic broadcast adaptive for weight change.  Section 6 discusses experiments serving to evaluate the performance of the proposed mechanism. Finally, Section 6 remarks on the conclusions drawn.

## 2. Related Work

This section reviews important attempts at applying data broadcasting and bandwidth allocation in mobile networks. This paper develops an analytical model to approximate the proposed TAB algorithm. This model makes it convenient to efficiently evaluate the mean access time of the generated broadcast program. Moreover, in light of the derived access time, both the index channel's optimum bandwidth allocation and the data channel's optimum bandwidth allocation are formulated.

In order to assess the feasibility and efficiency of our mechanism, we conducted several experiments. Results reveal that the proposed TAB algorithm performs well in terms of data-access efficiency. Moreover, the optimum bandwidth allocation yields a significant performance improvement in tune-in time. As a consequence, it can be seen from experimental results that putting the proposed mechanism into practice is entirely feasible.

In [5], this scheme assumes that data items are of equal sizes. In terms of practicality, it is not efficient to apply the broadcast disks to the varied-size data items. Moreover, it is hard for system developers to define the similarity of data popularity so as to partition data items into disks. The determination of the relative broadcast frequency for each disk is also imprecise. In this paper, we propose a TAB algorithm for varied-size data items to tackle the above drawbacks. The TAB algorithm grows a broadcast tree to determine the broadcast frequency of each data record. After that, we split the broadcast tree into some broadcast wood with similar sizes so as to place those data items in the broadcast cycle. The details are described clearly in Section 3.

**Figure 1:**
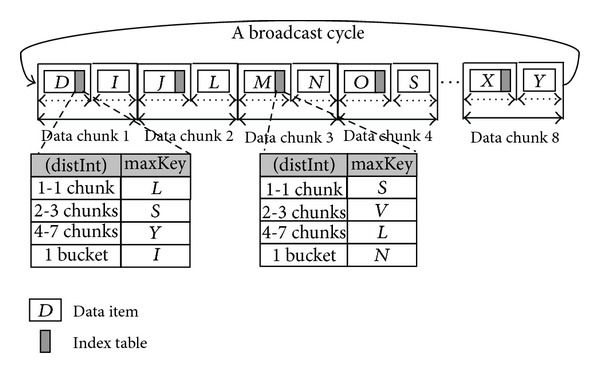
An example of an exponential index.

Xu et al. present exponential-index technology that enables some flexibility in trade-off between tune-in time and access latency [21]. As shown in Figure 1, the exponential index adopts a flat broadcast and disseminates data items in the ascending order of their identifiers. These researchers further group data items into a chunk and maintain one index table for each chunk. The number of entries in an index table is determined by the index base. Then the exponential-index technology can adjust the trade-off between access efficiency and energy consumption by tuning the index base and the chunk-size parameters.

Therefore, the bandwidth of an exponential index in broadcasting index information is only dominated by the index base and chunk size. The current study further examines the effects of data placement on broadcast programs and establishes an analytical model to derive the optimum bandwidth allocation for index packets and data elements. The performance comparison between our mechanism and the exponential index is demonstrated in Section 5.

**Figure 2:**
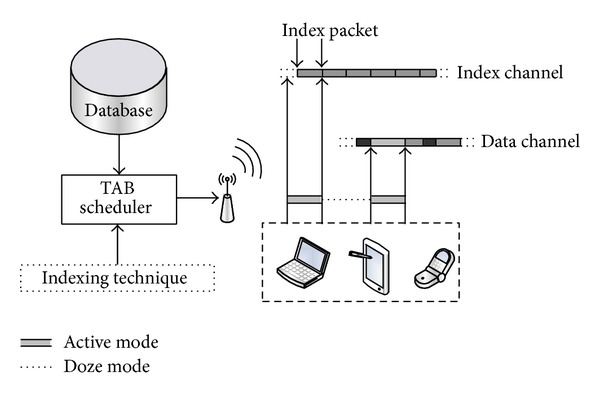
The sketch of our system architecture.

The system architecture in this paper is depicted in Figure 2. As shown in Figure 2, the proposed TAB algorithm schedules data items in a server's database to construct a broadcast program. According to the broadcast program, the system disseminates these data records periodically through the data channel. In addition, in order to reduce power consumption, some effective indexing techniques can generate index packets. Those index packets contain information for mobile nodes, such as data identifiers and the nearest data-appearance time. Index packets are broadcast through the index channel.

On the other hand, when a user submits queries to a mobile client, the mobile equipment first fetches an index packet from the index channel to get the arrival time of the target data. Then, the mobile device switches from the active mode to the doze mode for energy savings until the target item appears on the data channel [20]. After that, the mobile client downloads the target-data transaction so as to process the user's request.

Servers' databases maintain some auxiliary information for each data item. As depicted in Table 1, each data entry consists of three attributes: the data identifier, the data-access probability, and the data size. Addressing these factors, this paper proposes an efficient TAB algorithm to generate a skewed broadcast program. Afterwards, the paper proposes an analytical model to approximate the mean access time of our mechanism. We also derive the optimum index-channel and data-channel bandwidth allocations. Details are presented in the following sections.

## 3. Tree-Based Adaptive Broadcasting

In this section, we propose the TAB algorithm as a way to improve the performance of existing data-broadcasting mechanisms. According to the statistical probability of data access, the TAB algorithm broadcasts a significant number of copies for popular data in a broadcast cycle to diminish the average access time. In addition, the proposed algorithm balances the interbroadcast time of successive copies of a single packet of data even though data-item sizes can vary.

The notation for the TAB algorithm is summarized in Table 2. Consider the case in which a server's database contains *N* data items for broadcasting. The data-access probability and the data size for each data item are given as well. In terms of these factors, the TAB algorithm should construct an efficient broadcast program to reduce access time. In fact, the TAB algorithm can be split into three different steps: data-item reordering, broadcast-tree construction, and wood-size equalization. The details for each step are described as follows.

### 3.1. Data-Item Reordering

The first step of the TAB algorithm is to sort all data records in the database by their access frequency and size. More precisely, after performing the data-item reordering, we would get a broadcast cycle [*D*_1_, *D*_2_,…, *D*_*N*_] such that (1) Pr(*D*_*i*_) ≥ Pr(*D*_*j*_) and (2) if Pr(*D*_*i*_) = Pr(*D*_*j*_), and then *S*(*D*_*i*_) ≤ *S*(*D*_*j*_) for any integers *i* < *j* ≤ *N*. To reduce the average access time, it is beneficial to broadcast hotter data more frequently [27, 28]. Therefore, sorting data records from hottest to coldest can make it convenient to determine the broadcast frequency of each data item.

The second step is to determine the broadcast frequency (i.e., the number of replicates) for each data item. Note that the replication of a data record would reduce the access time of that data; however, the replication would lengthen the whole broadcast cycle and increase the access time of other records. Therefore, this paper proposes a broadcast-tree construction algorithm to balance the trade-off between these two factors.

**Figure 3:**
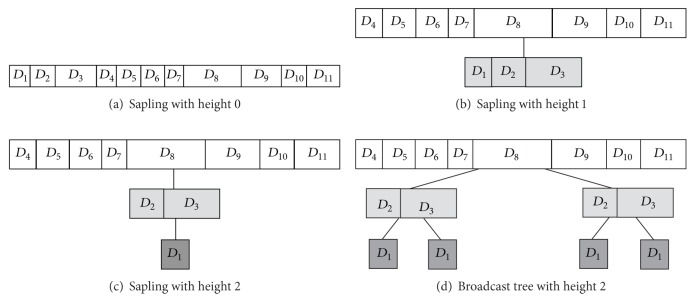
The scenario of the broadcast-tree construction.

As a matter of fact, the broadcast-tree construction yields broadcast trees in a top-down manner.  Figure 3 presents the scenario of the broadcast-tree construction. First of all, the broadcast-tree construction starts with the sorted data from the data-item reordering (Figure 3(a)). Then, after some evaluation, the algorithm iteratively moves data items with high access probabilities to the lower level so as to double their broadcast frequencies (Figures 3(b) and 3(c)). Finally, we can get a broadcast tree by copying the nodes at each level, resulting in a full binary tree as drawn in Figure 3(d).

### 3.2. Broadcast-Tree Construction

Once the broadcast tree is built, the broadcast frequency *n*_*i*_ for each data item *D*_*i*_ is determined as well. The number of replicates in the broadcast tree stands for the data's broadcast frequency. Thus, take the broadcast tree in Figure 3 as an example. In this case, we have *n*_1_ = 4, *n*_2_ = *n*_3_ = 2, and *n*_4_ = *n*_5_ = ⋯ = *n*_11_ = 1. Specifically, the criterion for estimating which data items should be moved to the next level is determined by the following theorem.

**Theorem 1 .**

**Figure 4:**
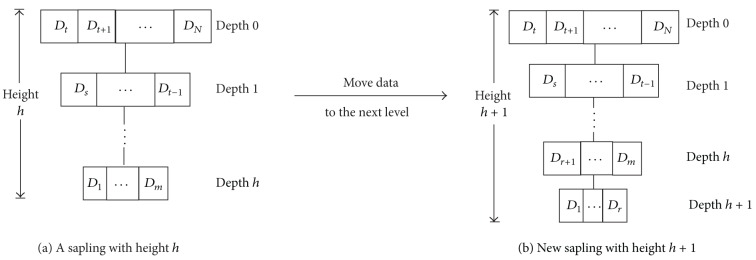
Move data items {*D*_1_,…, *D*_*r*_} to the next level.

Suppose that sapling *R* of height *h* has *m* data records at depth *h* (as shown in Figure 4(a)). Let *d*_*i*_ denote the depth of data item *D*_*i*_ in *R* and let *S*(*D*_*i*_) represent its data size (i.e., the broadcast frequency *n*_*i*_ = 2^*d*_*i*_^  and the broadcast length  *L* = ∑_*i*=1_^*N*^ *n*_*i*_ *S*(*D*_*i*_). Assume that the bandwidth of the data channel is *B*. Then the reduced average access time, achieved by moving data items *D*_1_,…, *D*_*r*_  (1 ≤ *r* ≤ *m*) to the next level *h* + 1, can be formulated by

(1) $$\begin{array}{c} \frac{L}{2^{h+2}B}\overset{r}{\underset{i=1}{\sum }}Pr\left(D_{i}\right)\\ -\frac{1}{B}\left(\overset{r}{\underset{i=1}{\sum }}\frac{Pr\left(D_{i}\right)}{4}+2^{h-1}\overset{N}{\underset{i=r+1}{\sum }}\frac{Pr\left(D_{i}\right)}{n_{i}}\right)\left(\overset{r}{\underset{i=1}{\sum }}S\left(D_{i}\right)\right). \end{array}$$

**Proof**

Consider the average access time before and after moving data to the next level. Figure 4 shows that, before data are moved, one can perform an approximate calculation of the mean access time *T*_BE_ by using the equation

(2) $$\begin{array}{c} T_{\text{BE}}=\overset{N}{\underset{i=1}{\sum }}\text{Pr}\left(D_{i}\right)\left(\frac{L_{\text{BE}}}{2n_{i}B}+\frac{S\left(D_{i}\right)}{B}\right), \end{array}$$

where *L*_BE_ denotes the current broadcast length. Likewise, after data are moved, the average access time *T*_AF_ can be estimated by means of the equation

(3) TAF=∑i=1rPr(Di)(LAF2·(2ni)B+S(Di)B) +∑i=r+1NPr⁡(Di)(LAF2niB+S(Di)B),

where the latter broadcast cycle length *L*_AF_ is equal to *L*_BE_ + ∑_*i*=1_^*r*^ *n*_*i*_ *S*(*D*_*i*_). Therefore, we can obtain the reduced access time

(4) TBE−TAF =∑i=1rPr(Di)4niBLBE  −(∑i=1rPr⁡(Di)4niB+∑i=r+1NPr⁡(Di)2niB)(∑j=1rnjS(Dj)) =LBE2h+2B∑i=1rPr⁡(Di)  −1B(∑i=1rPr⁡(Di)4+2h−1∑i=r+1NPr⁡(Di)ni)(∑j=1rS(Dj)).

According to Theorem 1, the constancy of bandwidth *B* facilitates the broadcast-tree construction procedure. The broadcast-tree construction starts with the sorted data elements from the data-item reordering.

Afterwards, we use Theorem 1 to determine the optimal cutpoint *c* for each level and move data records *D*_1_,…, *D*_*c*_ to the next floor so as to reduce the overall access time. In addition, note that the maximum height of the generated broadcast tree is limited by parameter *τ*. This factor can prevent the procedures shown in Algorithm 1 from taking too much execution time.

### 3.3. Wood-Size Equalization

Once the number of duplicates for each data item is obtained, we determine the replicated data placement in the broadcast cycle. Clearly, to achieve a better performance, the interbroadcast time of successive copies of data should be the same. However, it is known that such an optimum placement problem associated with the variant data sizes is an NP-complete problem [29]. Consequently, in this subsection we develop a wood-size equalization algorithm to place those data items in the broadcast cycle.

The functionality of the wood-size equalization is to split broadcast tree *I* of height *h* into 2^*h*^ pieces of broadcast wood (*w*_1_,…, *w*_2^*h*^_) with similar sizes. Actually, the wood-size equalization is a recurrence in structure. It splits the broadcast tree in a bottom-up manner. Given broadcast tree *I*, we first get 2^*h*^/2 broadcast woods by applying the wood-size equalization to the left subtree of *I* and get the other 2^*h*^/2 broadcast woods from the right subtree of *I*. Afterwards, the* root-cutting* procedure permits the distribution of the data in the root to these woods such that each broadcast wood has a similar size. The wood-size equalization can be stated as follows.

Basically, the root-cutting procedure adopts a greedy strategy to divide the root node. More precisely, the root-cutting procedure iteratively splits the data *a*_*i*_ with the largest data size from the root and attaches it to the minimum-size wood *w*_*c*_ until all the data in the root are allocated. Thus, we can summarize the root-cutting procedure in Algorithms 2 and 3.

**Figure 5:**
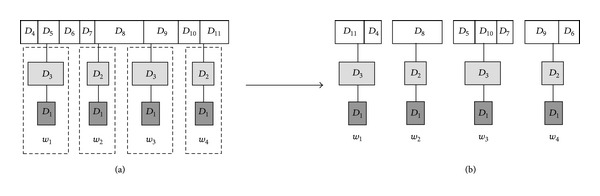
The scenario of the wood-size equalization.

An example of the proposed wood-size equalization is illustrated in Figure 5. Consider broadcast tree *I* of height 2 in Figure 3(d). In the beginning, we recursively apply wood-size equalization, resulting in the intermedium tree with four broadcast woods shown in Figure 5(a). After performing the root-cutting procedure, we get four individual broadcast woods as drawn in Figure 5(b).

**Figure 6:**
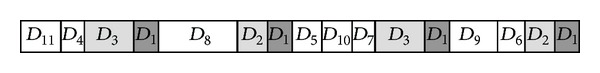
The generated broadcast program.

Upon completion of the wood-size equalization, one can obtain the broadcast cycle by sequentially broadcasting each wood from top to bottom. Therefore, in this case we can get the broadcast program as shown in Figure 6.

### 3.4. Complexity Analysis

In this subsection, the time complexity of the TAB algorithm is studied. Recall that the TAB algorithm contains three steps. The first step is the data-item reordering, which requires time complexity* O*(*N*log*N*) for sorting *N* data items. In addition, the second step, broadcast-tree construction, builds a broadcast tree having a height of at most *τ*. For each level, it takes at most* O*(*N*) time to determine the fittest cutpoint. And then, it requires* O*(2^*τ*^ *N*) time to expand from a sapling to a full binary tree. Finally, the wood-size equalization is a recurrence in structure, and it requires a time complexity of* O*(*τN*log*N *+  *τ*2^*τ*^ *N*). Besides, because the value *τ* is relatively insignificant for the large value of* N*, we concluded that the proposed TAB algorithm takes only a time complexity of* O*(*N*log*N*) in total.

## 4. Optimum Bandwidth Allocation

In this section, we develop an analytical model to approximate the average access time of the proposed TAB algorithm (Theorem 9). Afterwards, this analytical model helps derive the optimum bandwidth allocation for our system architecture and minimize the average access time (Theorem 10). The details are described as follows.

**Figure 7:**
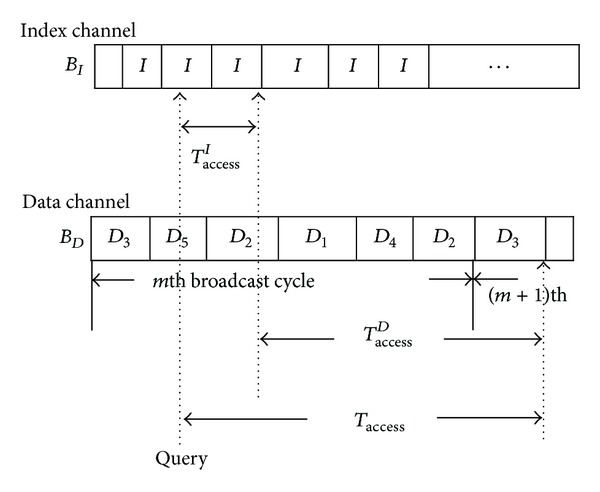
Sketch of the data-access time.

It can be seen from Figure 7 that the access time of the proposed TAB algorithm can be decomposed into two portions:* index-access time* and* data-access time*. When a user submits queries to mobile clients, the mobile device needs to read an index packet from the index channel. The time interval between the moment that the user sends a query and the moment that the mobile device gets one index packet is called the index-access time. Likewise, the data-access time is defined as the time interval between the moment that the mobile client finishes the index packet and the moment that the mobile device obtains the target data packet. As a result, by the above definitions, we have the following lemma.

**Lemma 2 .**

Let the random variable *T*_*access*_ denote the total access time of a query. And let the random variables  *T*_*access*_^*I*^  and  *T*_*access*_^*D*^  represent the index-access time and data-access time of the query, respectively. Thus, it is clear that

(5) $$\begin{array}{c} T_{access}=T_{access}^{I}+T_{access}^{D}. \end{array}$$

**Figure 8:**
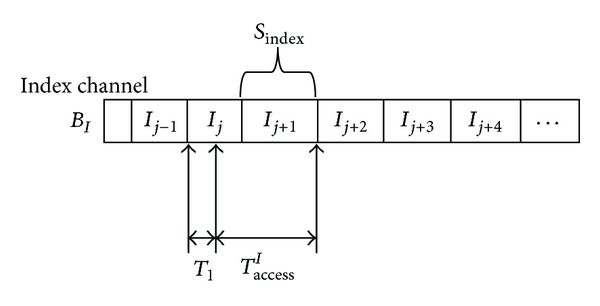
Sketch of the index-access time.

Therefore, according to Lemma 2, we know that the index-access time and data-access time should be calculated first to obtain the total access time. In order to get the index-access time, we consider the index channel as shown in Figure 8. It can be seen from Figure 8 that the index access time  *T*_access_^*I*^  is determined by the time the mobile device submits its query. Consequently, if we assume that a uniform distribution characterizes the duration of time extending from the starting point of the current index packets to the moment the mobile device submits its query [30], then the average index-access time can be arrived at by the following lemma.

**Lemma 3 .**

Let *S*_*index*_ represent the size of each index packet and let *B*_*I*_ denote the bandwidth of the index channel. Assume that the interval between the starting point of the current index packet and the moment the mobile client submits its query follows a uniform distribution over  [0, *S*_*index*_/*B*_*I*_). Then the average index-access time can be formulated as

(6) $$\begin{array}{c} E\left[T_{access}^{I}\right]=\overset{S_{index}/B_{I}}{\underset{0}{\int }}\left(2\cdot \frac{S_{index}}{B_{I}}-t\right)\cdot {\left(\frac{S_{index}}{B_{I}}\right)}^{-1}dt. \end{array}$$

**Proof**

Without loss of generality, we consider the case in which the mobile user submits a query during the *j*th index-packet broadcast time as in Figure 8. Let *T*_1_ denote the random variable representing the time interval between the starting point of the *j*th index-packet broadcast cycle and the moment that the mobile device submits its query. Thus, it is clear that the index-access time *T*_access_^*I*^ can be formulated as

(7) $$\begin{array}{c} T_{\text{access}}^{I}=\{\begin{array}{cc} \frac{S_{\text{index}}}{B_{I}} & \text{if}\;\;T_{1}=0\\ 2\frac{S_{\text{index}}}{B_{I}}-T_{1} & \text{if}\;0<T_{1}<\frac{S_{\text{index}}}{B_{I}}. \end{array} \end{array}$$

In addition, if we further assume that the random variable *T*_1_ follows a uniform distribution over  [0, *S*_index_/*B*_*I*_), then the mean index-access time can be computed by

(8) $$\begin{array}{c} E\left[T_{\text{access}}^{I}\right]=\overset{S_{\text{index}}/B_{I}}{\underset{0}{\int }}\left(2\cdot \frac{S_{\text{index}}}{B_{I}}-t\right)\cdot {\left(\frac{S_{\text{index}}}{B_{I}}\right)}^{-1}dt. \end{array}$$

On the other hand, since each data item *D*_*i*_ has its own access probability Pr(*D*_*i*_), the average data-access time can be expressed as a weighted summation of the average access time of all data items. In terms of mathematic form, the mean data-access time can be formulated as follows.

**Lemma 4 .**

Suppose that the server database contains *N* data items *D*_1_, *D*_2_,…, *D*_*N*_ for broadcasting. Furthermore, let Pr (*D*_*i*_) denote the access probability of the data item *D*_*i*_, for *i* = 1,2,…, *N*. Then the expected value of the data-access time can be expressed by

(9) $$\begin{array}{c} E\left[T_{access}^{D}\right]=\overset{N}{\underset{i=1}{\sum }}E\left[T_{access}\left(D_{i}\right)\right]\cdot \text{ Pr }\left(D_{i}\right), \end{array}$$

where *T*_*access*_(*D*_*i*_) stands for the random variable representing the access time of the specific data item *D*_*i*_.

**Figure 9:**
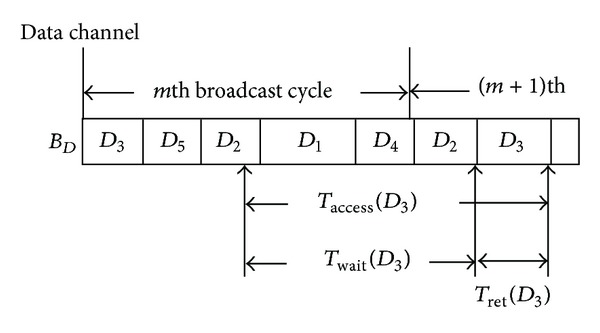
Sketch of the data-access time.

As a consequence, in order to obtain the average data-access time, we need to get the average access time for each data item first.  Figure 9 depicts the sketch of the data item *D*_3_'s access time. As shown in Figure 9, the access time of an arbitrary data item *D*_*i*_ can be further decomposed into two parts:* waiting time* and* retrieval time*. The waiting time of an arbitrary data item *D*_*i*_ is defined as the time interval between the moment the mobile device gets an index packet and the moment the data channel starts to broadcast the target data item *D*_*i*_. And the retrieval time represents the time interval during which the mobile equipment downloads the target data item. Thus, by the above definition, we have the following lemma.

**Lemma 5 .**

Let *T*_*wait*_(*D*_*i*_) denote the random variable representing the waiting time of the data item *D*_*i*_ and let *T*_*ret*_(*D*_*i*_) denote the retrieval time of the data item *D*_*i*_. Then we have

(10) $$\begin{array}{c} T_{access}\left(D_{i}\right)=T_{wait}\left(D_{i}\right)+T_{ret}\left(D_{i}\right). \end{array}$$

In addition, the retrieval time *T*_*ret*_(*D*_*i*_) can be determined by its data size *S*(*D*_*i*_) divided by the data channel bandwidth *B*_*D*_. That is,

(11) $$\begin{array}{c} T_{ret}\left(D_{i}\right)=\frac{S\left(D_{i}\right)}{B_{D}}. \end{array}$$

On the other hand, it is not easy to derive the waiting time *T*_wait_(*D*_*i*_) directly for some data items *D*_*i*_ because the proposed TAB algorithm broadcasts duplicates for those data items with high-access probability. Furthermore, the positions of the replicated data items in the broadcast cycle also determine the data items' waiting time. Therefore, in this paper, we introduce a specific *L*_*i*_[*k*]-function to represent the position of the *k*th replicate of the data item *D*_*i*_ in the broadcast cycle.

**Definition 6 .**

The length *L* of a broadcast cycle is defined as the total number of data bits in this broadcast cycle. And the term *L*_*i*_[*k*] is defined as the total number of broadcasted bits before broadcasting the *k*th replicate of the data item *D*_*i*_ in a broadcast cycle.

**Example 7 .**

Take the broadcast cycle depicted in Figure 6 as an example. In this case, we have the equations *L*_1_[1] = *S*(*D*_11_) + *S*(*D*_4_) + *S*(*D*_3_), *L*_1_[2] = *L*_1_[1] + *S*(*D*_1_) + *S*(*D*_8_) + *S*(*D*_2_), *L*_1_[3] = *L*_1_[2] + *S*(*D*_1_) + *S*(*D*_5_) + *S*(*D*_10_) + *S*(*D*_7_) + *S*(*D*_3_), and *L*_1_[4] = *L*_1_[3] + *S*(*D*_1_) + *S*(*D*_9_) + *S*(*D*_6_) + *S*(*D*_2_). The length of the broadcast cycle *L* is equal to *L*_1_[4] + *S*(*D*_1_).

**Figure 10:**
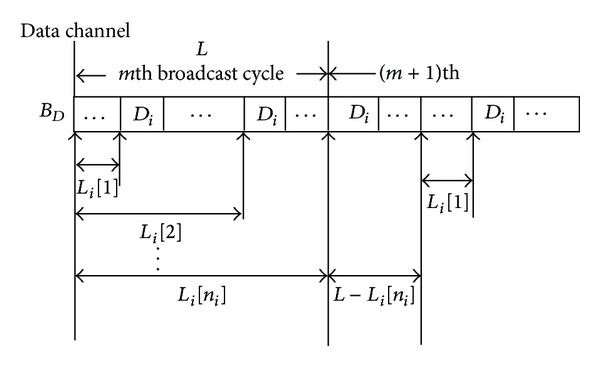
The broadcast deployment for data item *D*_*i*_ (*L*_*i*_-function).

As a result, we know that the *L*_*i*_[*k*]-function can help describe any broadcast program accurately. Consider the case in which, after this work performs the proposed TAB algorithm, the data item *D*_*i*_ has *n*_*i*_ duplicates in a broadcast cycle, and these *n*_*i*_ duplicates are located at *L*_*i*_[1], *L*_*i*_[2],…, *L*_*i*_[*n*_*i*_], respectively (see Figure 10). Subsequently, the following theorem can yield the data item's waiting time *T*_wait_(*D*_*i*_) relative to the proposed TAB scheme.

**Theorem 8 .**

Let *B*_*D*_ be the bandwidth of the data channel. Suppose that the broadcast program obtained by performing the TAB algorithm is given in terms of the *L*_*i*_[*k*]-function. Moreover, let the random variable T represent the time interval between the starting point of the current broadcast cycle and the moment that the mobile client starts to wait for the target data item. Then the random variable *T*_*wait*_(*D*_*i*_) can be formulated as

(12) $$\begin{array}{c} T_{wait}\left(D_{i}\right)=\{\begin{array}{cc} \frac{L_{i}\left[1\right]}{B_{D}}-T & if\;\;0\leq T<\frac{L_{i}\left[1\right]}{B_{D}}\\ \frac{L_{i}\left[k+1\right]}{B_{D}}-T & if\;\;\frac{L_{i}\left[k\right]}{B_{D}}\leq T<\frac{L_{i}\left[k+1\right]}{B_{D}}\\ & for\;\;k=1,2,\ldots ,n_{i}-1\\ \frac{L-T+L_{i}\left[1\right]}{B_{D}} & if\;\;\frac{L_{i}\left[n_{i}\right]}{B_{D}}\leq T<\frac{L}{B_{D}}. \end{array} \end{array}$$

Besides, if we assume that the random variable *T* follows a uniform distribution over  [0, *L*/*B*_*D*_), then the average waiting time *E*[*T*_*wait*_(*D*_*i*_)] can be further simplified as

(13) E[Twait(Di)]=12BDL[(L−Li[ni]+Li[1])2+∑k=1ni−1(Li[k+1]−Li[k])2].

**Proof**

Without loss of generality, we assume that the mobile client starts to wait for the target data item *D*_*i*_ in the *m*th broadcast cycle as in Figure 10. Since the data *D*_*i*_ is broadcast *n*_*i*_ times during a broadcast cycle, the mobile client retrieves the nearest replicate of the target *D*_*i*_ according to the entry time the mobile client starts to wait. So with time *T* being the time at which the mobile device starts to wait, we now consider three cases for calculating the waiting time.

*Case  1  *(0 ≤ *T* < *L*_*i*_[1]/*B*_*D*_). In this case, the mobile client starts to wait before the first replicate of the target item in the *m*th broadcast cycle is broadcast. Therefore, the waiting time can be obtained by

(14) $$\begin{array}{c} T_{\text{wait}}\left(D_{i}\right)=\frac{L_{i}\left[1\right]}{B_{D}}-T. \end{array}$$

*Case  2 *(*L*_*i*_[*k*]/*B*_*D*_ ≤ *T* < *L*_*i*_[*k* + 1]/*B*_*D*_, *k* = 1,2,…, *n*_*i*_ − 1). In this case, the mobile client retrieves the (*k* + 1)th replicate of the target data item in the *m*th broadcast cycle. Thus, the waiting time can be computed by

(15) $$\begin{array}{c} T_{\text{wait}}\left(D_{i}\right)=\frac{L_{i}\left[k+1\right]}{B_{D}}-T. \end{array}$$

*Case  3 *(*L*_*i*_[*n*_*i*_]/*B*_*D*_ ≤ *T* < *L*/*B*_*D*_). In this case, all the replicates of the target data item in the *m*th broadcast cycle were broadcast when the mobile device began to wait. Thus, the mobile client would download the first replicate of the target data in the (*m* + 1)th broadcast cycle. In other words, the waiting time can be formulated as

(16) $$\begin{array}{c} T_{\text{wait}}\left(D_{i}\right)=\frac{L-T+L_{i}\left[1\right]}{B_{D}}. \end{array}$$

On the other hand, if we further assume that the random variable *T* satisfies a uniform distribution over  [0, *L*/*B*_*D*_), then the expected value of the waiting time can be derived by

(17) E[Twait(Di)] =∫0Li[1]/BD(Li[1]BD−t)·(LBD)−1dt  +∑k=1ni−1∫Li[k]/BDLi[k+1]/BD(Li[k+1]BD−t)·(LBD)−1dt  +∫Li[ni]/BDL/BD(L−t+Li[1]BD)·(LBD)−1dt =12BDL[(L−Li[ni]+Li[1])2     +∑k=1ni−1(Li[k+1]−Li[k])2].

According to the above lemmas and theorems, the average access time can be computed as well. We now summarize the derivation of the average access time via the following theorem.

**Theorem 9 .**

The average access time *E*[*T*_*access*_] can be derived by

(18) E[Taccess]=3Sindex2BI+∑i=1N
Pr
⁡(Di)      ·{12BDL[(L−Li[ni]+Li[1])2           +∑k=1ni−1(Li[k+1]−Li[k])2]        +S(Di)BD}.

**Proof**

Based on Lemma 2, it is clear that

(19) $$\begin{array}{c} E\left[T_{\text{access}}\right]=E\left[T_{\text{access}}^{I}\right]+E\left[T_{\text{access}}^{D}\right]. \end{array}$$

Furthermore, after applying Lemmas 4 and 5 to the above equation, we get

(20) E[Taccess]=E[TaccessI]+∑i=1NPr(Di)       ·(E[Twait(Di)]+S(Di)BD).

Finally, Lemma 3 and Theorem 8 permit us to calculate the average access time *E*[*T*_access_] as follows:

(21) 3Sindex2BI+∑i=1NPr(Di)     ·{12BDL[(L−Li[ni]+Li[1])2          +∑k=1ni−1(Li[k+1]−Li[k])2]        +S(Di)BD}.

From Theorem 9, we can not only estimate the average access time of a query for any broadcast program, but also determine the optimum bandwidth allocation for both the index channel and the data channel. Consider the case in which the overall channel bandwidth for data broadcasting is *B*. Then, for the proposed TAB algorithm to achieve the minimum access time, the optimum bandwidth allocation is given by the following theorem.

**Theorem 10 .**

Let *B* denote the total bandwidth for data broadcasting. Then the optimum bandwidth settings necessary for index channel *B*_*I*_^*opt*^ and data channel *B*_*D*_^*opt*^ to achieve the minimum average access time can be formulated as

(22) $$\begin{array}{c} B_{I}^{opt}=\frac{\sqrt{3S_{index}}}{\sqrt{3S_{index}}+\sqrt{2\xi }}B,\;\;B_{D}^{opt}=\frac{\sqrt{2\xi }}{\sqrt{3S_{index}}+\sqrt{2\xi }}B, \end{array}$$

where

(23)   ξ=∑i=1N
Pr
(Di)  ·{12L·[(L−Li[ni]+Li[1])2      +∑k=1ni−1(Li[k+1]−Li[k])2]+S(Di)}.

**Proof**

According to Theorem 9, the average access time *E*[*T*_access_] is equal to

(24) $$\begin{array}{c} E\left[T_{\text{access}}\right]=\frac{3S_{\text{index}}}{2B_{I}}+\frac{\xi }{B_{D}}. \end{array}$$

Denote the estimation function Φ(*B*_*I*_, *B*_*D*_) by

(25) $$\begin{array}{c} \Phi \left(B_{I},B_{D}\right)=\frac{3S_{\text{index}}}{2B_{I}}+\frac{\xi }{B_{D}}. \end{array}$$

As a consequence, to minimize the average access time *E*[*T*_access_], we need to choose the values *B*_*I*_ and *B*_*D*_ to minimize the estimation function Φ(*B*_*I*_, *B*_*D*_) subject to the constraint *B*_*I*_ + *B*_*D*_ = *B*.

Assume that Γ(*B*_*I*_) = Φ(*B*_*I*_, *B* − *B*_*I*_). Then it is clear that the optimum bandwidth of the index channel *B*_*I*_^opt^ satisfies the equation

(26) $$\begin{array}{c} {\frac{d\Gamma (B_{I})}{dB_{I}}|}_{B_{I}=B_{I}^{\text{opt}}}=0. \end{array}$$

After substituting this into the estimation function, we have

(27) $$\begin{array}{c} {\frac{d\Gamma (B_{I})}{dB_{I}}|}_{B_{I}=B_{I}^{\text{opt}}}={\left(-\frac{3S_{\text{index}}}{2B_{I}^{2}}+\frac{\xi }{{\left(B-B_{I}\right)}^{2}}\right)|}_{B_{I}=B_{I}^{\text{opt}}}=0. \end{array}$$

That is,

(28) $$\begin{array}{c} B_{I}^{\text{opt}}=\frac{\sqrt{3S_{\text{index}}}}{\sqrt{3S_{\text{index}}}+\sqrt{2\xi }}B. \end{array}$$

Also, the optimum bandwidth of the data channel *B*_*D*_^opt^ can be arrived at through the following equation:

(29) $$\begin{array}{c} B_{D}^{\text{opt}}=B-B_{I}^{\text{opt}}=\frac{\sqrt{2\xi }}{\sqrt{3S_{\text{index}}}+\sqrt{2\xi }}B. \end{array}$$

## 5. Performance Evaluation

### 5.1. Simulation Environment

In order to assess the performance of the proposed system architecture, we conducted several experiments.  Table 3 shows the parameter settings in our experiments. We assumed that the number of total data records for broadcasting varied from 50 to 100. And each data item contained two attributes: data size and data-access frequency.

In our experiments, the data sizes followed a normal distribution with the mean varying from 50 KB to 150 KB and a variance of 900 KB^2^. The modeling of the data-access probabilities rested on the Zipf distribution with the parameter *θ* [31]. In other words, the probability of the data item *D*_*i*_ was assumed to be

(30) $$\begin{array}{c} \text{Pr}\left(D_{i}\right)=\frac{{\left(1/i\right)}^{\theta }}{\sum_{j=1}^{N}{\left(1/j\right)}^{\theta }}, \end{array}$$

where the value of skew factor *θ* ranged from 0.5 to 1.5.

We did not employ any indexing technology for the index channel in our system, and in this way we could realize the actual effects of the proposed method. The index packet size was assumed to be 128 bytes. Further, the total available bandwidth including the index and data channel was set to 80 KB/sec [3].

In addition to the proposed system architecture, we implemented a plain broadcast, broadcast disks, and an exponential index scheme for comparison. In line with the simulation model in [1], the broadcast-disk technology was implemented with three broadcast disks. And the relative frequencies between these disks were dominated by parameter Δ. More precisely, the broadcast frequency of the disk *i* was determined by *ref*_*freq*(*i*) = (3 − *i*)Δ + 1. In the experiments, we considered three kinds of broadcast disks schemes: Δ = 1, 2, and 3.

For each experiment, we generated five different datasets, each one containing 50~100 data records for broadcasting. In addition, for each data set, we generated 5,000 queries and calculated the corresponding access time and tune-in time to evaluate access efficiency and power conservation. The interarrival time of queries followed an exponential distribution with an arrival rate of *λ* = 1. The simulator and query generator were coded in MATLAB.

### 5.2. Experimental Results

This work applies average tune-in time and average access time as the performance measurement. It allows devices to power on when they need to access data so that these two values are lower than the ones gained by the other method. It means that this device can stay in sleep mode longer and save more energy.

**Figure 11:**
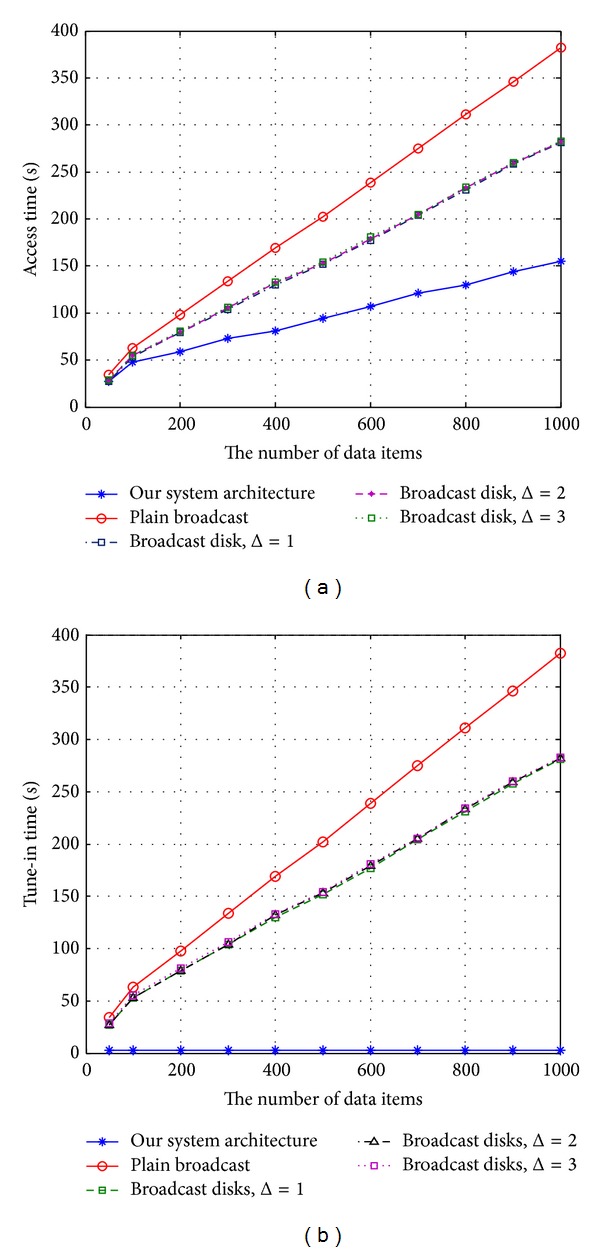
The average access time and tune-in time for various data-item numbers.

Figure 11 shows the average access time and tune-in time of different schemes with the number of data items varying from 50 to 100. In this experiment, we considered the case in which the Zipf parameter was set at 0.9 and the data-size generation process was a normal distribution with a mean of 100 KB and a variance of 900 KB^2^.

It can be seen from Figure 11(a) that even though we sacrificed some bandwidth to broadcast index packets, our mechanism still achieved a lower access time than the broadcast disks did. Furthermore, as presented in Figure 11(a), this phenomenon became more prominent as the number of data items increased.  Figure 11(b) shows the effects of our system on power conservation. As shown in Figure 11(b), the average tune-in time of our mechanism was lower than that of the exponential-index scheme. This finding demonstrates the importance of efficient bandwidth allocation in power conservation.

**Figure 12:**
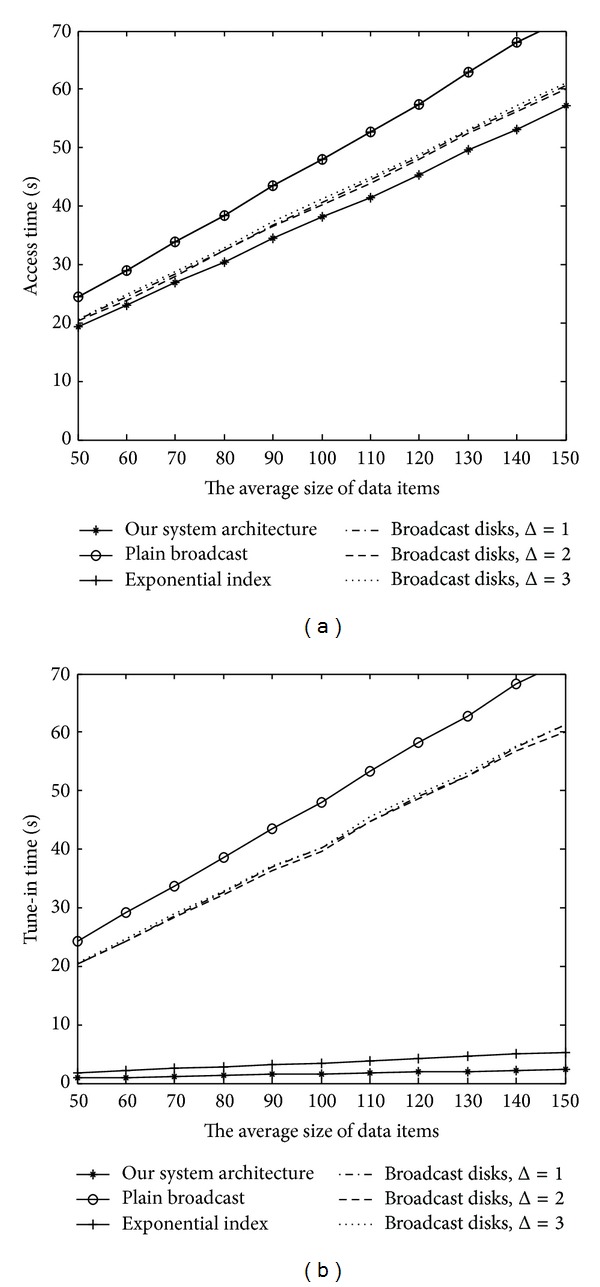
Performance comparison in various average data sizes.

Figure 12 presents our comparison of the access latency and tune-in time in various average data sizes. Without loss of generality, we considered the number of data records to be set at 75 and the Zipf parameter at 0.9 in this experiment. Furthermore, the data-size generation process followed a normal distribution with a mean ranging from 50 KB to 100 KB.

As shown in Figure 12(a), the average access latency of all schemes increased as the average data size increased. Nevertheless, our mechanism consistently outperformed all the other schemes for various data sizes. In addition, the slope of our mechanism proved to be smaller than that of the broadcast disks. A similar condition appeared in our comparison of the tune-in time. As depicted in Figure 12(b), because our system used the optimum bandwidth allocation, our mechanism could achieve a better performance in power conservation than the exponential index scheme.

**Figure 13:**
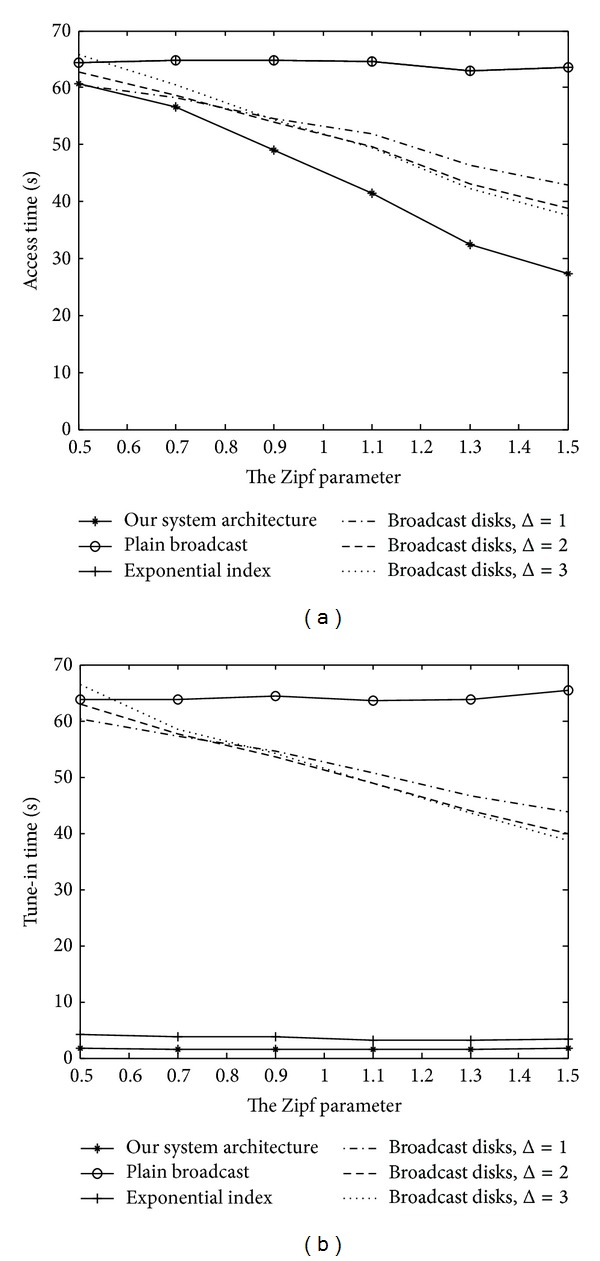
Performance comparison in various skew factors.

Regarding the effects of the skewness of the data-access probabilities, Figure 13 depicts our comparison of the average access time and tune-in time in various Zipf parameters. In this experiment, the Zipf parameter representing the skewness of data-access frequencies varied from 0.5 to 1.5. The number of data records was 100 and the data size had a normal distribution with a mean of 100 KB and a variance of 900 KB^2^.

It can be seen from Figure 13(a) that our mechanism significantly outperforms all the other schemes. Furthermore, the performance gain of our mechanism becomes more and more conspicuous as the value of the skew factor increases. This indicates that an efficient determination of the data-broadcast frequency is critical, especially the more skewed the data access is. In terms of power conservation, Figure 13(b) shows that the skew factor only slightly affects the tune-in time of our mechanism and the exponential index scheme.

**Figure 14:**
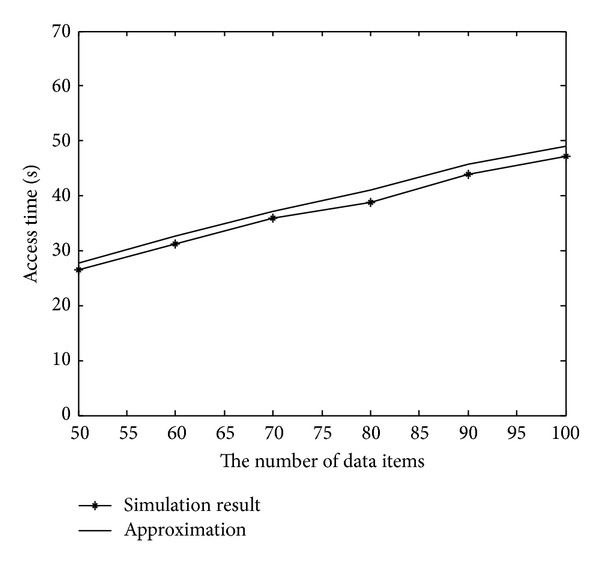
Average access time for experimental and analytical results.

Regarding the accuracy of our approximation model, Figure 14 presents our comparison between the analytic results and the experimental results. We obtained each point depicted in the curve of approximation by calculating the derived access time in Theorem 9. Each point shown in the curve of the simulation results represents the average access time of 5,000 data queries on various parameters. Again, Figure 14 shows that the curve of our approximation is close to that of the numerical results.

In conclusion, even though our system releases some bandwidth to broadcast index packets, our experimental results show that our mechanism exhibits better access latency than the plain broadcast and broadcast disks scheme do, especially when the data-access probabilities are skewed. In terms of power conservation, it is clear that our system can reduce much more tune-in time if a proper indexing technology is applied to our index channel. In addition, the numerical results of our experiments confirm the accuracy of the proposed approximation model.

## 6. Conclusion

Data broadcasting involves important data dissemination technology for accessing mobile services in wireless networks. In general, there are two main approaches to data broadcast, namely, push-based broadcast and on-demand broadcast [32]. Mobile Internet and mobile services that make use of mobile data are increasingly popular [33–35]. Among others, access efficiency and power conservation are two critical performance indexes for assessing the effectiveness of wireless communication systems. In this paper, we present a TAB algorithm to reduce the response time of mobile clients' requests. We provide an analytical model to measure the expected access latency of the generated broadcast program. This analytical model helps formulate the optimum bandwidth allocation for index and data channels. From the experimental results, it can be seen that our mechanism outperforms the existing data-broadcast schemes in terms of access time. Moreover, the optimum bandwidth allocation also brings about a significant improvement in energy conservation. Based on these advantages, it can be seen that the proposed mechanism is scalable and can feasibly increase the efficiency of data dissemination in broadcast-based systems.

## Figures and Tables

**Algorithm 1:**
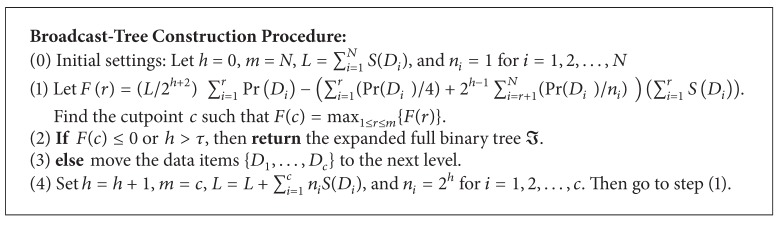


**Algorithm 2:**
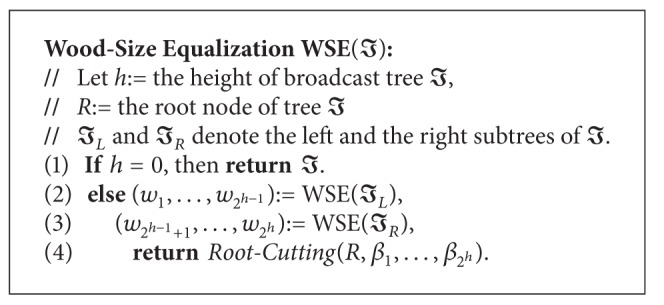


**Algorithm 3:**
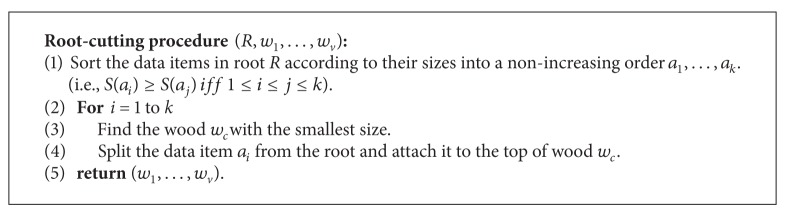


**Table 1:** Data structure in servers' databases.

Data identifier	Access probability	Size
*D*_1_	Pr(*D*_1_)	*S*(*D*_1_)
*D*_2_	Pr(*D*_2_)	*S*(*D*_2_)
*D*_3_	Pr(*D*_3_)	*S*(*D*_3_)
⋮	⋮	⋮

**Table 2:** Notation used in the TAB mechanism.

Notation	Definition
*N*	The number of data items
Pr(*D*_*i*_)	The access probability of data item *D*_*i*_
*S*(*D*_*i*_)	The size of data item *D*_*i*_
*n*_*i*_	The broadcast frequency of data item *D*_*i*_
*R*	Sapling
*I*	Broadcast tree
*h*	Tree height
*L*	The length of a broadcast cycle
*B*	The bandwidth of a broadcast channel
*τ*	Maximum tree height of a broadcast tree
*w*_*i*_	The *i*th broadcast wood

**Table 3:** Parameter setting.

Parameters	Values
The number of data items (*N*)	50~1000
Bandwidth (*B*)	80 KB/sec
Index packet size (*S*_index_)	128 bytes
The sizes of data items (*S*(*D*_*i*_))	Normal distribution (mean: 50~150 KB, variance: 900 KB^2^)
The access probabilities (Pr(*D*_*i*_))	Zipf distribution (*θ*= 0.5~1.5)
The number of requests	5000
Maximum height of broadcast tree	3
